# Folate Receptor 4-Expressing T cell Is Associated with Disease-Free Survival in Patients with Esophageal Squamous Cell Carcinoma

**DOI:** 10.1155/2022/4351949

**Published:** 2022-06-15

**Authors:** Xiangmei Zhang, Qi Zhao, Miao Cao, Xinhui Li, Xin Chen, Ming He, Yunjiang Liu, Jidong Zhao

**Affiliations:** ^1^Research Center, Fourth Hospital of Hebei Medical University, Shijiazhuang 050011, China; ^2^Hebei Provincial Key Laboratory of Tumor Microenvironment and Drug Resistance, Hebei Medical University, Shijiazhuang 050017, China; ^3^Department of Thoracic Surgery, Fourth Hospital of Hebei Medical University, Shijiazhuang 050011, China; ^4^Department of Breast Cancer Center, Fourth Hospital of Hebei Medical University, Shijiazhuang 050011, China; ^5^Cancer Institute, Fourth Hospital of Hebei Medical University, Shijiazhuang 050011, China

## Abstract

**Background:**

Folic acid receptor 4 (FR4) significantly downregulates the expression of regular T cells (Treg) and improves the effect of chemotherapy and PD-1/PD-L1 inhibitors. However, the FR4 expression in squamous cell carcinoma (ESCC) remains unclear.

**Methods:**

Patients with primary ESCC who visited our hospital between 1^st^ February 2012 and 30^th^ September 2016 were enrolled in this study. FR4 expressions in ESCC patients were detected by immunohistochemistry staining, and the association with clinical characteristics and the overall survival (OS) or disease-free survival (DFS) was analyzed.

**Results:**

One hundred and forty-eight qualified cases of ESCC patients were retrieved, including 34 females. Ninety-four cases had lymph node metastasis (63.51%), 104 patients received adjuvant therapy (70.27%), and the rate of FR4 positive was 67.57% (100/148). Among FR4 positive patients, 75 cases received adjuvant therapy, and patients who received chemotherapy were significantly better than that of patients who did not receive chemotherapy. In patients with FR4 negative expression, 48 cases received adjuvant therapy, which was significantly worse than that of patients who did not receive chemotherapy.

**Conclusions:**

Postoperative adjuvant chemotherapy prolonged the survival in FR4 positive ESCC patients, whereas adjuvant therapy in patients with FR4 negative needs to be further improved.

## 1. Introduction

Esophageal cancer is a common malignant tumor. Esophageal squamous cell carcinoma (ESCC) is the major form of esophageal cancer, accounting for more than 90% in China. One of its prominent clinical features is the early occurrence of metastasis, while the prognosis of patients with advanced ESCC is poor, with a 5-year survival rate of only 16-20% [1]. Although radical esophagectomy improves the survival rate of ESCC patients, the recurrence rate is still as high as 27%-52%, limiting its clinical efficacy [2, 3]. Chemotherapeutic drugs such as cisplatin, 5-fluorouracil (5-FU), and paclitaxel for ESCC exhibit obvious limitations in clinical outcomes [4]. Currently, immunotherapeutic monoclonal antibodies offer hope for the treatment of ESCC. With the release of clinical trial results such as CheckMate 577, Keynote-590, Keynote-180, and Keynote-181, PD-1/PD-L1 antibodies have been developed as a first-line treatment for advanced ESCC [5]. However, poor overall response, susceptibility to drug resistance, and lack of predictive biomarkers remain challenges for current immunotherapy for ESCC.

Recent study indicated that ESCC tissues express abundant immune cells, and immune escape mediated by regulatory T cells (Treg) not only promotes the development of ESCC but also mediates the development of therapeutic tolerance [6]. Inhibition of the development of primary Tregs to effector Tregs significantly reduces the occurrence of tumor immune escape and improves the efficacy of chemotherapy and other tumor treatments. Therefore, identification of effective markers of primary Treg is critical for the ESCC therapy. Folate receptors (FRs) are cell surface glycosylphosphatidylinositol- (GPI-) anchored glycoproteins. There are four forms of FRs, folic acid receptors 1-4 (also known as FR-*α*, FR-*β*, FR-*γ*, and FR-*δ*), which are mainly expressed in the placenta and kidney [7]. The expression of FRs increases markedly once epithelial cells develop malignancies such as in ovarian and lung cancers [8]. In recent years, folate receptor 4 (FR4) has been found to be specifically expressed in tumor-associated macrophages (TAMs) and Tregs to regulate the immunosuppressive functions [9]. In this study, the FR4 expression in ESCC patients was determined, and the association between FR4 with clinical characteristics and the disease-free survival (DFS) was analyzed, with the aim to identify predictive biomarkers for ESCC therapy.

## 2. Materials and Methods

### 2.1. Specimen and Patients

A total of 148 patients with primary ESCC treated at the Fourth Hospital of Hebei Medical University between 1^st^ February 2012 and 30^th^ September 2016 were enrolled in this study. Inclusion criteria are as follows: (1) adult cases with esophageal squamous cell cancer diagnosed by postsurgical histopathology; and (2) at least 5 years of follow-up after surgery. Exclusion criteria are as follows: (1) combined with other types of cancer; and (2) withdrawal during follow-up or incomplete data. This study was approved by the Institutional Ethnics Committee of the Fourth Hospital of Hebei Medical University, China. Informed consent was obtained at the time of patient admission. Authors were able to identify information about individual participants during data collection and analysis.

### 2.2. Immunohistochemistry (IHC) Staining

The expressions of FR4 in tumor tissues were detected by immunohistochemistry staining. Paraffin-embedded specimens were sectioned (4 *μ*m). IL-22 (clone ab18499, 1 : 100 dilution, Abcam, Cambridge, UK) and IL-22R1 (clone ab5984, 1 : 100 dilution, Abcam, Cambridge, UK) expression was quantified by using the Histo-score (*H*-score) system. The intensity (0 = no staining, 1^+^ = weak, 2^+^ = moderate, 3^+^ = strong) was scored and the percentage of staining tumor cells in the whole section was evaluated. The *H*-score was calculated using the following formula: (3 × percentage of cells with strong staining) + (2 × percentage of cell with moderate staining) + (1 × percentage of cells with weak staining). IL-22 expression was classified into two groups according to a cutoff *H*-score of 100 [10]. For CD4 (clone BM4263, 1 : 50 dilution, Boster, Wuhan, China), CD8 (clone BM4379, 1 : 30 dilution, Boster, Wuhan, China), FOXP3 (clone ab20034, 1 : 50 dilution, Abcam, Cambridge, UK), and CD68 (clone BA3639, 1 : 100 dilution, Boster, Wuhan, China) immunohistochemistry staining, expression in nuclei of lymphocytes was defined as positive. The density of these infiltrating immune cells was categorized as high or low relative to the median of 11 cells/0.0625 mm^2^ as the cutoff value within the slide at high power (×40 objective lens).

The paraffin-embedded sections were treated in xylene and rehydrated through an ethanol gradient. Antigen retrieval was performed by heating slides for 15 minutes at 95°C in citrate buffer (pH 9.0). Endogenous peroxidase activity was blocked with 3% H2O2 for 30 minutes followed by blocking with 5% goat serum (except goat antihuman primary Abs). Specimens were incubated with primary antibody at 4°C overnight. The nuclei were counterstained with hematoxylin (Dako). Rabbit IgG and goat IgG were used as isotype controls.

For evaluation of CD68 immunohistochemistry staining, expression in nuclei of peritumor immune cells infiltrated in stromal tissues was defined positive. The density of these infiltrating immune cells was categorized as high or low relative to the median of 11 cells/0.0625 mm^2^ as the cutoff value within the slide at high power (×40 objective lens).

### 2.3. Statistical Analysis

Statistical analysis was performed by using the SPSS version 21.0 (IBM SPSS Statistics, Armonk, NY, USA). Data were presented as mean ± SD. The associations between FR4 expression and immune cells infiltration with pathologic characteristics were examined using Chi-square statistical tests. OS and DFS were estimated by the Kaplan-Meier method and compared with log-rank test. The correlation between FR4 expression and CD68 infiltration with clinical survival was evaluated by COX regression in both univariate and multivariate analyses. All tests were two-sided, and *P* values <0.05 were considered significant.

## 3. Results

### 3.1. Expression of FR4 in Tumor-Infiltrating Lymphocytes (TILs) of ESCC

In this study, a total of 148 ESCC cases were retrieved and analyzed. The baseline characteristics are shown in Table 1. There were 114 male and 34 female patients. In addition, 94 patients had lymph node metastasis (63.51%), and 104 patients received adjuvant therapy (70.27%).

**Figure 1:**
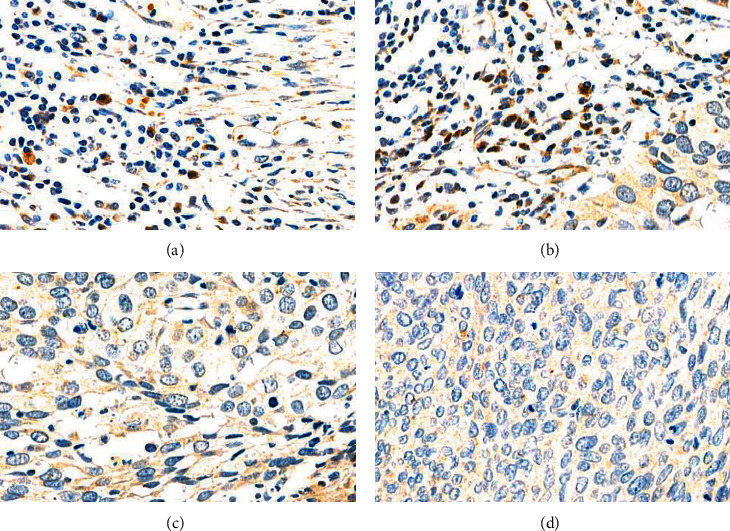
Expression of folate receptor 4 (FR4) in tumor-infiltrating lymphocytes (TILs) of esophageal squamous cell carcinoma (ESCC) patients from this study with IHC. FR4+ TILs (a and b) FR4-, (c) IgG isotype control, and (d) in ESCC patients.

This study retrospectively investigated tumor specimens from 148 ESCC patients. The expression of FR4 in tumor tissues was detected by IHC (Figure 1). The FR4 positive rate was 67.57% (100/148). No significant differences in general characteristics between FR4 positive and negative groups were observed (Table 2). As shown in Figure 1, FR4 expression was restricted in tumor-infiltrating lymphocytes (TILs).

**Figure 2:**
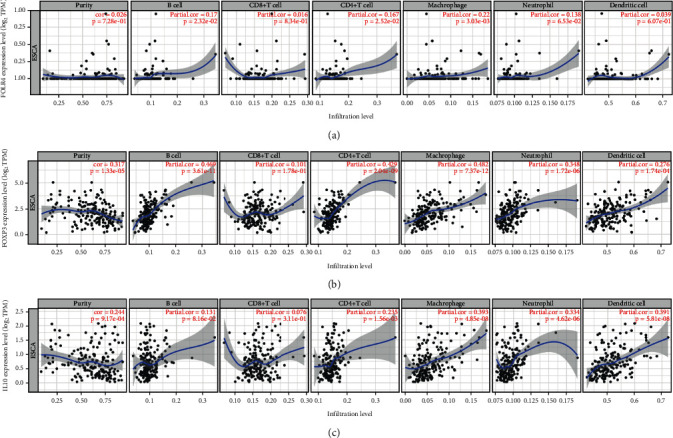
Correlation of Foxp3, IL-10, and folate receptor 4(FR4) expression with immune infiltration in esophageal squamous cell carcinoma (ESCC) patients from Tumor IMmune Estimation Resource (TIMER) database. FR4 expression has a special positive correlation with infiltration of CD4+ T cells and macrophages. (a) Foxp3 expression has a positive correlation with infiltration of CD4+ T cells, B cells, macrophages, neutrophils, and dendritic cells. (b) IL-10 expression has a positive correlation with infiltration of CD4+ T cells, macrophages, neutrophils, and dendritic cells (c) in ESCC patients.

We analyzed the data in the TIMER database (http://timer.cistrome.org/) [11] and found that Foxp3 gene expression level in ESCC tissues was positively correlated with the degree of B cell infiltration, CD4 positive T cells, macrophages, neutrophils, and dendritic cells (Figure 2(a)). The expression level of IL-10 gene was positively correlated with the infiltration degree of CD4 positive T cells, macrophages, and dendritic cells in tumors (Figure 2(b)). The FR4 level was only positively correlated with the degree of invasion of CD4 positive T cells and macrophages in tumors (Figure 2(c)). FR4 is more specific as an indicator of the degree of infiltration of CD4 positive T cells and macrophages in ESCC.

### 3.2. FR4 Expression in TILs Was Associated with Poor Prognosis

**Figure 3:**
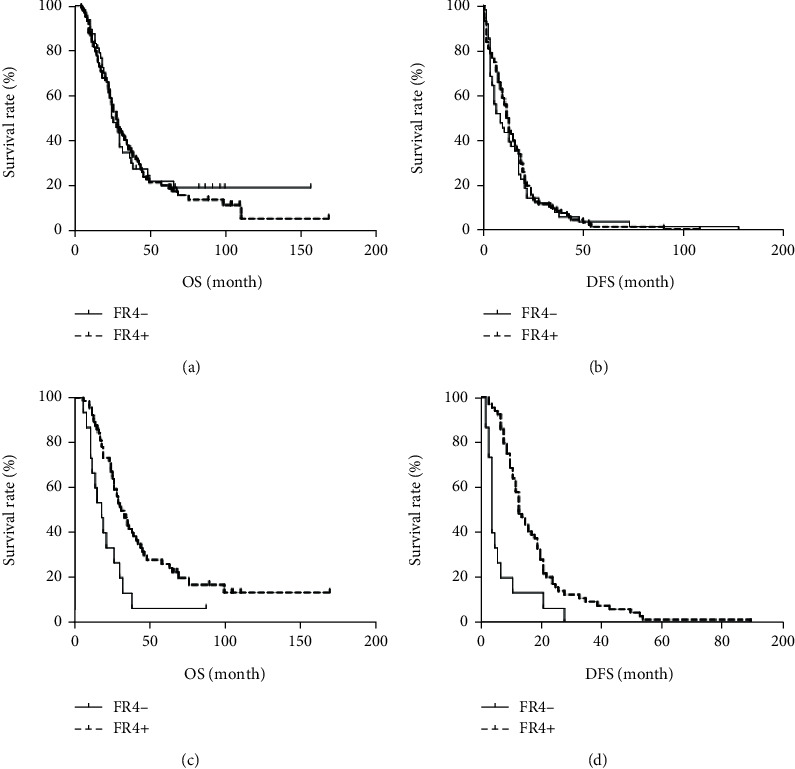
Folate receptor 4 (FR4) expression as predictor of overall survival in esophageal squamous cell carcinoma (ESCC) patients underwent adjuvant chemotherapy. The Kaplan-Meier estimate was used to perform the survival analysis from the date of the initial diagnosis and the log-rank test was used to compare the subgroup survival. (a and b) Overall survival (OS) and disease-free survival (DFS) in patients with or without FR4 expression. (c and d) OS and DFS in patients who underwent adjuvant chemotherapy with or without FR4 expression. FR positive (*n* = 100) and FR negative (*n* = 48).

The patients were followed up for 27 ± 28.38 months. The OS of FR4 positive and negative patients was 28 ± 27.72 and 25 ± 30.02 months, respectively, and there was no significant difference between them. Among FR4 positive patients, 75 received adjuvant therapy, and survival analysis showed that the patients who received chemotherapy (OS 30 ± 29.40 months; DFS 15 ± 13.45 months) were significantly better than those who did not receive chemotherapy (OS 18.5 ± 22.04 months; DFS 4 ± 12.5 months). Among patients with negative FR4 expression, 48 received adjuvant therapy, and survival parameters (OS 19 ± 15.10 months; DFS 7 ± 4.4 months) were significantly lower than those of patients (OS 25 ± 23.20 months; DFS 13 ± 11.15 months) who did not receive chemotherapy (Figure 3).

### 3.3. PD-L1 Expression Was Higher in FR4 Positive Patients

**Figure 4:**
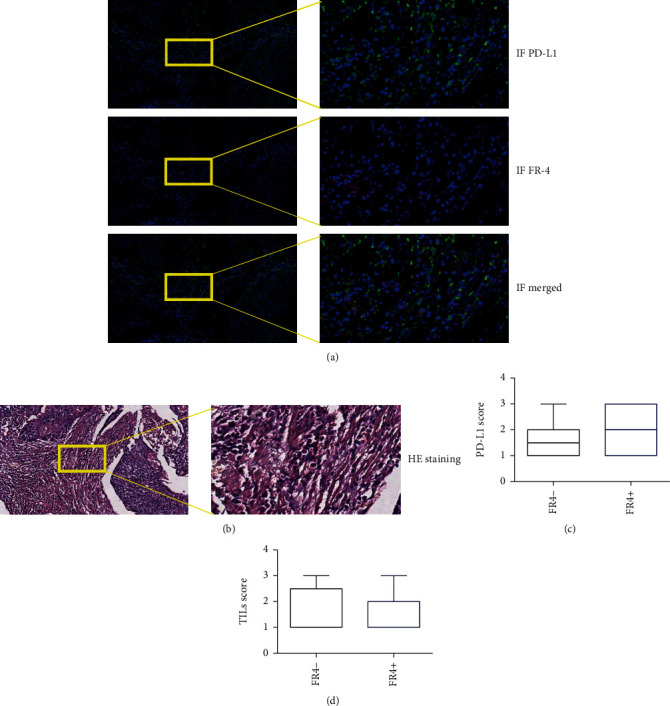
Expression of folate receptor 4 (FR4) and PD-L1 in ESCC patients from this study with immunohistochemistry staining, TIL scores with HE staining. FR4 expression (red) and PD-L1 expression (green) in samples of ESCC (a), TILs in samples of ESCC (b), and difference of PD-L1 scores (c) and TIL scores (d) in subgroups with FR4 expression.

The relationship between folate receptor 4 positive cells and tumor PD-L1 expression and tumor lymphocyte infiltration index was further analyzed. Tumor specimens were stained with immunofluorescence double staining (Figure 4(a)) and HE staining (Figure 4(b)). The results showed that patients with PD − L1 expression ≤ 1% accounted for 35.3% of the total patients, patients with PD − L1 expression > 1% but <50% accounted for 41.2%, and PD − L1 expression ≥ 50% accounted for 23.5% of patients. PD-L1 expression was consistent with the expression regions of FR4 positive cells, but there was no significant difference in PD-L1 expression between FR4 positive and negative patients (*P* = 0.17) (Figure 4(c)). The results of HE staining showed that the degree of TIL in ESCC was different, and patients with evaluation analysis ≤10% accounted for 44.1% of the total patients, 32.4% of patients with PD-L1 expression >10% while <50%, and PD-L1 expression ≥50% accounted for 23.5% of patients. There was no significant difference in TIL index between FR4 positive and negative patients (*P* = 0.66) (Figure 4(d)).

**Figure 5:**
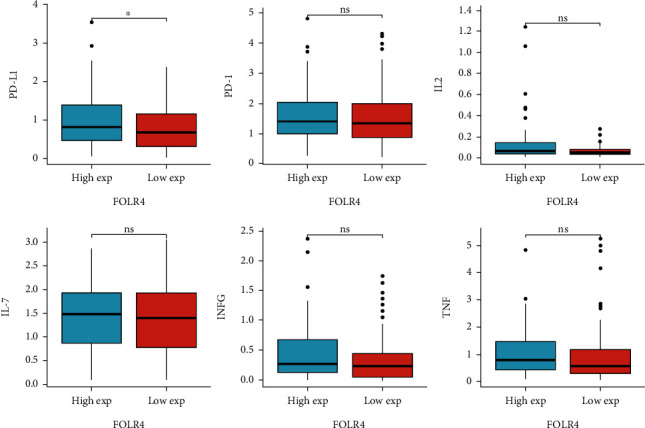
Correlation of folate receptor 4 (FR4) expression with PD-L1, PD-1, IL-2, IL-7, IFN-*γ*, and TNF-*α* in ESCC patients from TCGA database. FR positive (*n* = 100) and FR negative (*n* = 48).

To further reveal the relationship between FR4 positive cells and tumor immune microenvironment, we used 90 ESCC data from the TCGA public database to analyze PD-1, PD-L1, IL-2, IL-7, and levels of IFN-*γ* and TNF-*α*. PD-L1 expression was significantly higher in FR4 positive patients compared with negative patients (Figure 5). There were no significant differences in PD-1, IL-2, IL-7, and IFN-*γ* and TNF-*α* expression between FR4 positive and negative patients (Figure 5).

## 4. Discussion

In this study, FR4 expression in 148 ESCC patients was determined by IHC and analyzed the association between clinical characteristics and the OS and DFS, aiming at identifying the predictive biomarkers for ESCC immunotherapy.

Our results suggested that FR4 expression levels influence the efficacy of patients receiving adjuvant therapy. The survival time of patients with high FR4 expression was significantly prolonged after adjuvant chemotherapy. Patients' immune status affects the response to comprehensive therapy, and the proportion of T cell subsets and tumor-associated macrophages can predict the efficacy of comprehensive therapy for esophageal cancer [10]. Regulatory T cells in tumors are mostly marked by Foxp3. Previous study have shown that its predictive value in different malignant tumors is inconsistent, indicating that different subtypes of Treg have different effects on tumors [12]. As a marker of initial Treg, when FR4 is highly expressed in tumors, it indicates that the immune status in tumor tissues is different, and adjuvant chemotherapy may alter the immune escape status of tumors [9].

Data from TIMER for ESCC showed that FR4 was specifically positively correlated with Th cells and macrophages in tumor tissues. FR4 is one of the surface receptors of folic acid. Recent study demonstrated that FR4 is constitutively highly expressed on mouse CD4^+^CD25^+^ natural regulatory T cells (Treg) [13]. High expression of FR4 on Tregs can be used in combination with CD4 and CD25 to differentiate Tregs from other types of T cells. Since FR4 is a cell surface marker, Tregs were classified and detected in combination with anti-CD4 and/or CD25. Our analysis also showed the specificity of FR4 for CD4+ T cells and macrophages. Tregs with high FR4 expression are considered markers of TGF-*β*-induced and natural Tregs [14], while Foxp3 high expression is a marker of efferent Treg, and the expression of FR4 in efferent Treg is significantly downregulated [14]. Some subtypes of macrophages also express FRs including FR4. Macrophages collected from the peritoneal cavity of mice were stimulated by injection of normal saline, mercaptoacetate, yeast polysaccharide, and heat-inactivated or live bacteria. In addition to normal saline, F4/80+ macrophages in the peritoneal cavity of mice injected with other stimuli expressed FRs [15]. Furthermore, these macrophages had the expression of activation markers (CD80, CD86, and LY-6C/G), and when macrophages lacked the expression of these activation markers, there was little or no FR expression. FR4 can be used as a biological marker of Treg and macrophage subsets and functional status in tissues.

The expression region of FR4 overlapped with that of PD-L1 to a certain extent [16]. Our data showed that there was a certain correlation between FR4 and PD-L1, and this correlation has also been identified in TCGA database. Previous studies have shown that FR4 can be used as a therapeutic target for tumors, and the combined inhibition of PD-1 and FR4 improves the therapeutic effect of tumors. However, there is no definite evidence about the interaction between PD-L1 and FR4, and further *in vitro* experiments are needed to verify.

In conclusion, our study suggested that FR4 can be used as a marker of postoperative adjuvant chemotherapy benefit in esophageal squamous cell carcinoma, and FR4 is a marker of Treg and TAM, which has a certain correlation with PD-L1 expression. FR4 has potential to be a therapeutic target for esophageal cancer.

## Figures and Tables

**Table 1:** Baseline characteristics of the patients studied.

	Number	Rate
Gender		
Male	114	77.03
Female	34	22.97
Age	58 ± 7.56	
Tumor local		
Upper	9	6.08
Middle	101	68.24
Lower	38	25.68
Tumor size	5 ± 2.05	
pTNM stage		
1	5	3.38
2	59	39.86
3	80	54.05
4	4	2.70
Lymph node metastasis		
Negative	54	36.49
Positive	94	63.51
Differentiation grade		
Well	89	60.13
Poor	53	35.81
Undifferentiated	6	4.06
Folate receptor 4		
Negative	48	32.43
Positive	100	67.57

**Table 2:** Baseline characteristics of the patients between two groups.

	Folate receptor-		Folate receptor+		*P* value
	Number	Rate	Number	Rate	
Gender					0.99
Male	37	77.08	77	77	
Female	11	22.92	23	23	
Age	60 ± 5.57		58 ± 7.07		0.08
Tumor local					0.99
Upper	3	6.25	6	6	
Middle	33	68.75	68	68	
Lower	12	25	26	26	
Tumor size	5.5 ± 2.17		5 ± 1.83		0.14
pTNM stage					0.21
1	1	2.08	4	4	
2	25	52.09	34	34	
3	21	43.75	59	59	
4	1	2.08	3	3	
Lymph node metastasis					0.10
Negative	22	45.83	32	32	
Positive	26	54.17	68	68	
Differentiation grade					0.69
Well	29	60.42	60	60	
Poor	18	37.5	35	35	
Undifferentiated	1	2.08	5	5	
Adjuvant chemotherapy					
Yes	16	33.33	75	75	1.08E-06
No	32	66.67	25	25	

## Data Availability

The data used to support the findings of this study were supplied by the Institutional Ethnics Committee of the Fourth Hospital of Hebei Medical University under license and so cannot be made freely available. Requests for access to these data should be made to Dr. Yunjiang Liu, e-mail: lyj818326@hebmu.edu.cn.
